# Translation and validation of Cerebral Palsy Quality of Life Questionnaire-Teen in Hong Kong Chinese population [CP QoL-Teen (HK)]

**DOI:** 10.1007/s00431-023-04845-0

**Published:** 2023-02-09

**Authors:** Shirley P. C. Ngai, L. Y. Wong, Vitti W. K. Poon, Candice Y. C. Poon, Beverley P. H. Yiu, Teresa P. S. Wong, C. P. Chow

**Affiliations:** 1 Department of Rehabilitation Sciences, The Hong Kong Polytechnic University, Hong Kong SAR, China; 2 Child Assessment Service, Department of Health, Hong Kong SAR, China

**Keywords:** CP, QoL, Self-report, Proxy-report

## Abstract

Cerebral palsy (CP) is an early onset, non-progressive, neuromotor disorder. Adolescence is the transition from childhood to adulthood when changes in physical and emotional aspects and self-perception occur further imposing an impact to quality of life (QoL) in individuals with CP. Cerebral Palsy Quality of Life (CP QoL) Teen is a questionnaire examining different domains of QoL for adolescents with CP. This study is aimed at translating and validating self-report and proxy-report CP QoL-Teen (HK). Prior approval of translation has been obtained. Forward and backward translations were performed following standardized translation procedures. Participants and their caregivers were asked to complete self-report and proxy-report CP QoL-Teen (HK), and Child Health Questionnaire (CHQ). Internal consistency and test–retest reliability were assessed by Cronbach’s alpha and intraclass correlation coefficient (ICC), respectively. Concurrent validity was evaluated by Spearman’s rank correlation between subscales of CP QoL-Teen (HK) and CHQ as well as expanded and revised version of Gross Motor Function Classification System (GMFCS-E&R). Ninety-six participants completed the study. Of these, twenty participants completed CP QoL-Teen (HK) twice. Cronbach’s *α* of CP QoL-Teen (HK) ranged from 0.84 to 0.95 suggesting excellent internal consistency. Moderate to excellent test–retest reliability were demonstrated in all subscales of CP QoL-Teen (HK) (self-report: ICC = 0.46–0.8; proxy-report: ICC = 0.40–0.72, *p* < 0.05). Weak to moderate association between subscales of CP QoL-Teen (HK) and CHQ (self-report: *r*_s_ = 0.24–0.61; proxy-report: *r*_s_ = − 0.41–0.60) was reported.

*Conclusion*: This study showed that CP QoL-Teen (HK) has good psychometric properties. It is a valid and reliable tool to assess quality of life of adolescents with CP.

**Table:** 

**What is Known:** • *Cerebral Palsy Quality of life-Teen (CP QoL-Teen) is a validated tool with strong psychometric properties and clinical utility in gauging the QoL in adolescents with CP during their transition from childhood to adulthood when changes in physical and emotional aspects and self-perception occur. Yet, a locally validated tool is lacking in measuring the QoL for adolescents with CP in Hong Kong.* **What is New:** • *The Chinese translated version CP QoL-Teen (HK) is a valid and reliable tool to assess quality of life of adolescents with CP tailoring to the local cultural and social background with good psychometric properties being demonstrated.*

**What is Known:**

• *Cerebral Palsy Quality of life-Teen (CP QoL-Teen) is a validated tool with strong psychometric properties and clinical utility in gauging the QoL in adolescents with CP during their transition from childhood to adulthood when changes in physical and emotional aspects and self-perception occur. Yet, a locally validated tool is lacking in measuring the QoL for adolescents with CP in Hong Kong.*

**What is New:**

• *The Chinese translated version CP QoL-Teen (HK) is a valid and reliable tool to assess quality of life of adolescents with CP tailoring to the local cultural and social background with good psychometric properties being demonstrated.*

## Introduction

Cerebral palsy (CP) is an early onset, non-progressive, neuromotor disorder that affects developing fetal or infant brain imposing impacts on the development of movement and posture thereby causing functional limitation [1]. Its prevalence is 2.11 per 1000 live births (95%CI 1.98–2.25) [1] globally and 1.3 per 1000 children (aged 6–12) in Hong Kong [2]. There are various levels of functional limitations secondary to the abnormalities in muscle tone, strength, and coordination. Other than physical impairment, disturbances to sensation, visual perception, cognition, communication, behavior, and other medical conditions are also commonly reported.

In conceptualizing children’s health, apart from the traditional emphasis on considering the physical and anatomical disturbances the health condition had impacted on the patient’s body function, the International Classification of Functioning, Disability and Health-Child and Youth Version (ICF-CY) highlights the need of interpreting the impact of the disease and treatment effectiveness in a broader view [3]. This suggests that there is a need to analyze how it affects the activity, participation, and quality of life (QoL) of the patient.

Adolescence is the transitional period from childhood to adulthood when changes in physical, emotional, and self-perception occur. As a chronic neuro-disability, the effect of CP manifests differently throughout life span [4]. Patients with CP are very well taken care of in the medical sector during their early childhood. It is well established that gross motor function improves in all children with CP up to the age of 7 years, reaching plateau till adolescence [5]. Ambulatory children with CP are very likely to experience deterioration in function secondary to physical growth and musculoskeletal abnormalities, which results in the deterioration of mobility. Apart from such medical needs, they also have to face psychological, social, educational, and vocational challenges. As such, it is essential to have a comprehensive assessment tool to evaluate their quality of life. Yet, the function and quality of life beyond childhood, i.e., during adolescence and adulthood, are less well studied.

Cerebral Palsy Quality of Life (CP QoL) was developed by clinicians and researchers at the University of Melbourne and the Royal Children’s Hospital. It covers the age ranges of 4–12 (i.e., CP QoL-Child) [6] and 13–18 (i.e., CP QoL-Teen) [7]. Both CP QoL-Child and CP QoL-Teen were reported to be validated tools with strong psychometric properties and clinical utility [7, 8] in evaluating different aspects of quality of life among people with CP. CP QoL-Teen, an extension of CP QoL-Child, evaluates the change in quality of life during the transitional stage from childhood to adulthood [7]. The CP QoL-Teen has adolescent self-report version and primary caregiver proxy-report version. The self-report version consists of 72 items evaluating the adolescents’ life in “general wellbeing and participation,” “communication and physical health,” “school wellbeing,” “social wellbeing,” and “feelings about functioning” while the proxy report version has 88 items with the additional items to evaluate two extra subscales of “family health” and “access to service” [7]. The higher the score, the better the quality of life is represented. With knowledge that the measure of quality of life is subjective, it is often difficult to evaluate it for children and adolescents, especially those with illness, and therefore requiring the completion of proxy-report from parents [9]. However, inconsistent findings regarding agreement and disagreement on self- and proxy-ratings on the quality of life have been reported [10]. To measure the impact of the disease and interventions for patients with CP, a local validated tool on both self-report and proxy-report versions would be essential. This is to help clinicians evaluate the quality of life for adolescents with CP and better understand their needs for service planning.

CP QoL-Teen has been translated into different languages, yet there is still no Chinese version for the local population to date. Hence, this study is aimed at translating and validating CP QoL-Teen (HK), both self-report and primary caregiver proxy-report, on evaluating the quality of life of adolescents with CP.

## Materials and method

### Participants

Adolescents with confirmed diagnosis of CP were recruited from rehabilitation departments, normal schools, special schools, and children assessment center scattered across different regions in Hong Kong. Those who had neurodegenerative disease, psychiatric illness, or did not fully understand the questions during the screening were excluded. The primary caregivers of the eligible participants who were literate were invited to join and complete the primary caregiver report form. Ethical approval was obtained from the institutional review board of the involved university. Study details were explained, and informed consent was obtained from the participants’ guardian/parents prior to the study.

### Translation and cross-cultural adaptation of CP QoL-Teen (self-report and proxy-report)

Permission was obtained from original author to translate adolescents self-report and primary caregiver proxy-report of CP QoL-Teen. The translation process was based on the guidelines described by Beaton and co-workers [11]. The original English version of CP QoL-Teen (self-report and primary caregiver proxy-report) was translated to Chinese by a professional translator (C1) and a healthcare professional (C2) who were proficient in both Chinese and English. The two translated Chinese versions (C1 and C2) were then reviewed and combined to formulate a compromised forward translation version (CP QoL-Teen_C12) by an independent healthcare professional. Thereafter, this combined version (CP QoL-Teen_C12) undergone a backward translation to English by an independent professional translator (E1) and an independent healthcare professional (E2) before it was compiled by another healthcare professional. The compiled backward translation version was taken for comparison against the original English version to examine if any discrepancies existed. Detailed documents and records were kept for further discussion. The final version was critically reviewed by an interdisciplinary team consisting of academicians as well as clinical experts including pediatricians, physiotherapists, and clinical psychologists who had high proficiency in both Chinese and English and extensive clinical experience to work with children/adolescents with CP and developmental disorders. The team critically appraised the final version taking into consideration of the cultural needs to ensure its suitability to use in clinical settings. The self-report and proxy-report versions of CP QoL-Teen (HK) were evaluated by 10 adolescents with CP and their primary caregivers, as a pilot study, before its full implementation. Comments on clarification and/or revision of the translated text received were worked upon, and revised versions of CP QoL-Teen (HK) in self-report and proxy-report were finalized.

### Procedures

All eligible and consented participants were asked to fill in the self-report CP QoL-Teen (HK) while those with severe impairment requiring assistance were interviewed to complete at the referred centers/schools. Basic demographic information such as age, height, weight, and study year were collected onsite. The primary caregivers of the participants were given information pack [with the CP QoL-Teen (HK) primary caregiver (proxy-report), Child Health Questionnaire (proxy-report), and a short survey about their family related issues]. All these were returned by postage.

### Sensitivity

The sensitivity of CP QoL-Teen (HK) was assessed by evaluating the floor and ceiling effects where percentage of minimal and maximal score reached in each subscale.

### Test–retest reliability

A sample of 20 participants and their primary caregivers were invited to complete the self-report and proxy-report versions of CP QoL-Teen (HK) twice, with at least one week apart, to examine the test–retest reliability of CP QoL-Teen (HK).

### Validity of CP QoL-Teen (HK)

The concurrent validity of CP QoL-Teen (HK) was assessed by comparing the subscales of CP QoL-Teen (HK) and the subscales of the generic health related QoL questionnaire, i.e., Child Health Questionnaire (CHQ), and motor function by using the expanded and revised version of the Gross Motor Function Classification System (GMFCS-E&R) as listed below:

### Child Health Questionnaire (CHQ) Chinese version

The Child Health Questionnaire (CHQ) is a generic health-related QoL questionnaire assessing the functional health and well-being of children/adolescents aged 5 to 18 years old [12]. The CHQ-child (CHQ-CF-87) is self-reported by children/adolescents while the parent/proxy-report (CHQ-PF50) is reported by their parents. Both measures the unique physical and psychosocial concepts. The scale used ranges from 0 to 100, where the higher the score, the better the quality of life. Validated Chinese versions of CHQ-CF87 and CHQ-PF50 [13] were used in this study to examine the concurrent validity of CP QoL-Teen (HK).

### Expanded and revised version of Gross Motor Function Classification System (GMFCS-E&R)

The Gross Motor Function Classification (GMFCS) was originally developed to classify the gross motor function for children with CP using a 5-level classification system or children aged under 12 with good interrater reliability (ICC = 0.5 to 0.75 depending on the age) [14]. The expanded and revised version of Gross Motor Function Classification System (GMFCS-E&R), a 5-level classification, was developed in 2008 to classify the motor function of children/adolescents aged 12 to 18 with content validity [15] and interrater reliability reported [16].

### Statistical analysis

All data is presented as mean ± standard deviation (SD) unless otherwise indicated. Internal consistency was assessed by Cronbach’s *α* [17] with a value ≥ 0.7 indicating high reliability, with Cronbach’s *α* if deleted to assess item redundancy. Spearman correlation was used to examine the concurrent validity between adolescents (self-report) and primary caregiver (proxy-report) of CP QoL-Teen (HK). A range of 0.25 to 0.5 indicates a fair relationship; 0.5 to 0.75 indicates a moderate to good relationship; ≥ 0.75 indicated a good to excellent relationship [18]. Test–retest reliability was assessed by the intraclass correlation coefficient (ICC) between CP-QOL-Teen subscale scores measured in the first and second test. Agreement between the self-report and proxy report subscale scores was assessed by ICC. All data analyses were conducted using IBM® SPSS® Statistical software version 28 (IBM Corp. USA) with significant level set at *p* < 0.05.

## Results

### Characteristics of participants and primary caregivers

Ninety-six adolescents (mean age of 15.8 ± 2.1, male: 53.6% and female: 46.9%) with confirmed diagnosis of CP (spastic: 89.6%, dyskinesia: 6.3%, mixed: 3.1%, ataxic: 1%), recruited from territory-wide normal schools as well as all special schools serving the physical impairment population, participated this study. About one-third of the participants were born at gestation weeks < 30 weeks. Participants were distributed across different levels of GMFCS-E&R (level I: 54.2%; level II: 19.8%; level III: 19.8%; level IV: 6.3%; level V: 0%). Majority of the participants studied at secondary school (junior form: 43.8%; senior form: 39.6%) while others studied at primary school (16.7%). Most of them received normal education curriculum (77.1%) while some received adjusted curriculum (i.e., tailored and individualized education curriculum to meet with their developmental and education needs in association with the intellectual disability) (22.9%) (Table 1).

**Table 1:** Demographic data of adolescents and their primary caregivers

	Response category	Mean ± SD or frequency (%)
Adolescents (*n* = 96)		
Age (yr)		15.8 ± 2.1
Height (cm) (*n* = 93)		1.57 ± 0.1
Weight (kg) (*n* = 93)		49.9 ± 13.1
BMI (kg/m2) (*n* = 93)		20.1 ± 4.4
Gender (*n*, %)	Male	51 (53.6%)
	Female	45 (46.9%)
Type of CP (*n*, %)	Spastic	86 (89.6%)
	Ataxic	1 (1%)
	Dyskinesia	6 (6.3%)
	Mixed	3 (3.1%)
GMFCS-E&R level (*n*, %)	I	52 (54.2%)
	II	19 (19.8%)
	III	19 (19.8%)
	IV	6 (6.3%)
	V	0 (0%)
Type of study curriculum (*n*, %)	Normal level	74 (77.1%)
	Adjusted level	22 (22.9%)
Education level (*n*, %)	Primary school	16 (16.7%)
	Secondary school (junior form)	42 (43.8%)
	Secondary school (senior form)	38 (39.6%)
Primary caregiver (*n* = 91)		
Primary caregiver (*n*, %)	Father	18 (19.8%)
	Mother	71 (78%)
	Grandparent	2 (2.2%)
	Not to disclose	0 (0%)
Age category (*n*, %)	< 30	0 (0%)
	30–39	10 (11%)
	40–49	37 (40.7%)
	50–59	35 (38.5%)
	> 60	6 (6.6%)
	Not to disclose	3 (3.3%)
Education level (*n*, %)	None	4 (4.4%)
	Primary school	14 (15.4%)
	Secondary school	61 (67.0%)
	Tertiary education	8 (8.8%)
	Not to disclose	4 (4.4%)
Family monthly income (in HKD)	< $10,000	22 (24.2%)
	$10,000–$20,000	29 (31.9%)
	$20,001–$30,000	13 (14.3%)
	$30,001–$40,000	7 (7.7%)
	> $40,001	6 (6.6%)
	Not to disclose	14 (15.4%)
Gestation weeks (*n*, %)	< 30	32 (35.2%)
	31–38	35 (38.5%)
	> 38	18 (19.8%)
	Not to disclose	6 (6.6%)

Data were presented as mean ± standard deviation (SD) or frequency (*n*, %)

*BMI* body mass index, *CP* cerebral palsy, *GMFCS-E&R* expanded and revised version of Gross Motor Function Classification System

Ninety-one primary caregivers of the 96 adolescents participated and returned the questionnaire. Of these, mothers (78%) were the key caregiver followed by fathers (19.7%) and grandparents (2.2%). Approximately 75.8% of them had completed secondary education or above (Table 1). Regarding the home financial status, well above half of the family (56%) had a monthly income lower than the reported median monthly household income in Hong Kong [19].

### CP QoL-Teen (HK) self-report and proxy-report and concurrent validity

The mean scores of subscales of CP QoL-Teen, both self-report and proxy-report, were presented in Table 2. In general, there was a decreasing trend in the scores across the level of GMFCS-E&R in self-report form, despite insignificant. While for proxy-report form, no observable trend was detected. However, one-way ANOVA revealed that there were significant between-group differences detected in the subscale of feelings about functioning and access to service (*p* < 0.05). Post hoc analysis using the Bonferroni adjustment showed that the difference detected between GMFCS-E&R levels II and III was the subscale of access to service only. As aforementioned, majority of adolescents received education using normal curriculum (77.1%). When comparing the subscale scores reported by adolescents and their primary caregivers taking these curricula, no between-group differences were detected in all subscales of the self-report and five out of seven subscales of the proxy-report (Table 3). However, significantly lowered scores were found in the subscales of “feelings about functioning” (by − 10.8 unit, *p* = 0.01) and “access to service” (− 7.9 unit, *p* = 0.047) reported by primary caregivers of adolescents receiving adjusted curriculum when compared with those receiving normal curriculum.

**Table 2:** CP QoL-Teen (HK) subscale score across the levels of GMFCS-ER

Subscale (no. of items)	All		GMFCS-ER								ANOVA *p* value	
			I		II		III		IV			
	Self-report (*n* = 96)	Proxy-report (*n* = 91)	Self-report (*n* = 52)	Proxy-report (*n* = 52)	Self-report (*n* = 19)	Proxy-report (*n* = 15)	Self-report (*n* = 19)	Proxy-report (*n* = 18)	Self-report (*n* = 6)	Proxy-report (*n* = 6)	Self-report	Proxy-report
General wellbeing and participation (21)	68.2 ± 14.7	62.6 ± 12.9	70.0 ± 16.3	62.8 ± 12.7	64.6 ± 10.1	64.5 ± 9.4	66.9 ± 14.7	59.1 ± 17.4	68.5 ± 10.9	66.3 ± 4.8	0.56	0.55
Communication and physical health (16)	65.4 ± 14.6	62.5 ± 11.8	67.4 ± 16.8	62.0 ± 11.5	61.2 ± 3.4	64.2 ± 5.7	63.0 ± 14.7	60.5 ± 16.4	69.0 ± 9.1	68.5 ± 8.9	0.33	0.49
School wellbeing (8)	69.5 ± 16.0	67.2 ± 12.3	70.0 ± 17.5	65.8 ± 14.0	67.8 ± 11.8	67.1 ± 9.1	70.3 ± 17.4	69.2 ± 9.5	67.9 ± 12.5	72.7 ± 10.7	0.95	0.52
Social welling (7)	69.2 ± 16.7	71.6 ± 13.3	70.1 ± 17.1	71.3 ± 14.1	64.0 ± 16.3	68.4 ± 15.2	70.1 ± 16.0	74.3 ± 10.0	74.4 ± 15.9	75.0 ± 10.9	0.45	0.58
Feelings about functioning (5)	73.2 ± 19.2	58.9 ± 15.7	76.5 ± 18.9	67.6 ± 14.7	73.2 ± 15.9	61.0 ± 19.5	68.9 ± 22.2	58.6 ± 17.9	57.9 ± 13.3	58.6 ± 12.5	0.10	0.03*
Family health	N/A	63.6 ± 16.7	N/A	62.1 ± 14.1	NA	54.0 ± 17.4	NA	55.1 ± 19.1	NA	54.7 ± 8.3	NA	0.17
Access to service	N/A	66.9 ± 15.7	NA	67.1 ± 15.5	NA	74.1 ± 16.6^	NA	59.1 ± 140^	NA	70.4 ± 12.4	NA	0.047*

Data were presented as mean ± standard deviation (SD). Each subscale score of CP QoL-Teen ranged from 0 to 100—the higher the score, the better the quality of life being represented

*GMFCS-E&R* expanded and revised version of Gross Motor Function Classification System

^*^ indicates *p* < 0.05 in one-way ANOVA; ^ denotes *p* < 0.05 during post hoc analysis using Bonferroni adjustment

**Table 3:** CP QoL-Teen (HK) subscale score—comparison between adolescents undertaking normal and adjusted curriculum of education

Subscale (no. of items)	All		Normal curriculum		Adjusted curriculum		Independent *t*-test Mean difference (95%CI)	
	Self-report (*n* = 96)	Proxy-report (*n* = 91)	Self-report (*n* = 74)	Proxy-report (*n* = 71)	Self-report (*n* = 22)	Proxy-report (*n* = 20)	Self-report	Proxy-report
General wellbeing and participation (21)	68.2 ± 14.7	62.6 ± 12.9	68.2 ± 14.6	63.4 ± 11.9	68.3 ± 15.3	59.9 ± 16.2	− 0.13 (− 7.23 to 6.97)	3.48 (− 3.01 to 9.97)
Communication and physical health (16)	65.4 ± 14.6	62.5 ± 11.8	65.5 ± 14.3	63.3 ± 11.4	65.2 ± 15.8	59.7 ± 12.9	0.37 (− 6.79 to 7.33)	3.57 (− 2.34 to 9.49)
School wellbeing (8)	69.5 ± 16.0	67.2 ± 12.3	69.7 ± 15.0	66.2 ± 13.2	68.8 ± 19.6	70.7 ± 7.5	0.91 (− 6.87 to 8.67)	− 4.5 (− 9.13 to 0.06)
Social welling (7)	69.2 ± 16.7	71.6 ± 13.3	68.5 ± 15.7	71.6 ± 14.6	71.5 ± 19.8	71.7 ± 9.2	− 3.0 (− 11.06 to 5.05)	− 0.09 (− 6.83 to 6.65)
Feelings about functioning (5)	73.2 ± 19.2	58.9 ± 15.7	74.6 ± 16.6	66.0 ± 16.0	68.5 ± 26.0	55.3 ± 16.6	6.0 (− 6.04 to 18.11)	10.8* (2.64 to 18.87)
Family health	N/A	63.6 ± 16.7	N/A	59.9 ± 15.6	N/A	55.4 ± 15.8	N/A	4.5 (− 3.55 to 12.53)
Access to service	N/A	66.9 ± 15.7	N/A	68.6 ± 15.7	N/A	60.8 ± 14.5	N/A	7.9* (0.09 to 15.6)

Data were presented as mean ± standard deviation (SD). Each subscale score of CP QoL-Teen ranged from 0 to 100—the higher the score, the better the quality of life being represented

^*^ indicates *p* < 0.05 in independent *t*-test

When exploring the association between CP QoL-Teen and GMFCS-E&R, “feelings about functioning” was the only subscale being found to be negatively associated with the increased levels of GMFCS-E&R in self-report (*r*_s_ = − 0.26, *p* = 0.011) and proxy-report (*r*_s_ = − 0.28, *p* = 0.008) suggesting the higher the category of GMFCS-E&R, the lower the CP QoL-Teen score being reported by both adolescents and primary caregivers.

For self-report CP QoL-Teen (HK), fair to moderate association between subscales of CP QoL-Teen and CHQ-CF87 was observed (Table 4). In brief, subscales of (1) general wellbeing and participation, (2) communication and physical health, and (3) feeling about functioning were positively correlated with all subscales of CHQ-CF87 (*r*_s_ = 0.24 to 0.56, *p* < 0.05) except bodily pain (*p* > 0.05) (Table 4). Subscales of (4) school wellbeing and (5) social wellbeing of CP QOL were correlated with all subscales of CHQ-CF87 (*r*_s_ = 0.21 to 0.61, *p* < 0.05) except physical health and bodily pain (*p* > 0.05) (Table 4). For proxy-report of CP-QoL-Teen (HK), fair to moderate correlation between subscales of CP QoL-Teen and CHQ-PF50 was observed. In brief, subscales of (1) general wellbeing and participation and (2) feelings about functioning were significantly correlated with all subscales of CHQ-PF50 (*r*_s_ = − 0.26 to 0.59, *p* < 0.05, Table 5). Subscales of (3) communication and physical and (4) family were correlated with nearly all subscales of CHQ-PF50 (*r*_s_ =  − 0.41 to 0.60, *p* < 0.04, Table 5) except change of health (*p* > 0.05). Other subscales such as school wellbeing, social wellbeing, and access to services were correlated with some of the subscales of CHQ-PF50 (Table 5).

**Table 4:** Spearman rho correlation between subscales of CP QoL-Teen (HK) and CHQ (self-report)

CP QoL-Teen (HK)–self-report (*n* = 96)	CHQ-CF87													
	Global health	Physical function	Role-emotional	Role- behavior	Physical health	Bodily pain	Behavior	Global behavior	Mental health	Self esteem	Change of health	General health	Family activity	Family cohesion
General wellbeing and participation	0.33	0.34	0.46	0.30	0.24	0.15	0.34	0.46	0.49	0.48	0.50	0.31	0.26	0.39
*p* value	0.001**	0.001**	< 0.001**	0.003**	0.021**	0.154	< 0.001**	< 0.001**	< 0.001**	< 0.001**	< 0.001**	0.002**	0.012*	0.15
Communication and physical health	0.52	0.39	0.46	0.32	0.24	0.18	0.45	0.49	0.53	0.46	0.56	0.42	0.30	0.51
*p* value	< 0.001**	< 0.001**	< 0.001**	0.001**	0.021*	0.08	< 0.001**	< 0.001**	< 0.001**	< 0.001**	< 0.001**	< 0.001**	< 0.001**	< 0.001**
School wellbeing	0.34	0.27	0.46	0.33	0.15	0.19	0.44	0.41	0.46	0.51	0.47	0.28	0.36	0.48
*p* value	0.001**	0.009**	< 0.001**	0.001**	0.136	0.061	< 0.001**	< 0.001**	< 0.001**	< 0.001**	< 0.001**	0.005**	< 0.001**	< 0.001**
Social wellbeing	0.25	0.18	0.46	0.25	0.19	0.08	0.43	0.42	0.61	0.58	0.47	0.21	0.31	0.5
*p* value	0.016*	0.09	< 0.001**	0.014*	0.065	0.446	< 0.001**	< 0.001**	< 0.001**	< 0.001**	< 0.001**	0.04*	0.003**	< 0.001**
Feeling about function	0.35	0.48	0.39	0.35	0.27	0.13	0.50	0.37	0.42	0.38	0.40	0.26	0.37	0.29
*p* value	< 0.001**	< 0.001**	< 0.001**	0.001**	0.008**	0.2	< 0.001**	< 0.001**	< 0.001**	< 0.001**	< 0.001**	0.012*	< 0.001**	0.005**

^*^ indicates *p* < 0.05; ** indicates *p* < 0.01

**Table 5:** Spearman rho correlation between subscale of CP QoL-Teen (HK) and CHQ (proxy-report)

CP QoL-Teen (HK)–proxy-report (*n* = 91)	CHQ-PF50														
	Global health	Physical functioning	Role-emotion	Role –physical	Bodily pain	Behavior	Comparing with others, your child’s behavior	Mental health	Self-esteem	General health	Change of health	Parent impact—emotional	Parent impact—time	Family activity scale	Family cohesion
General wellbeing and participation	0.38	0.29	0.33	0.28	0.23	0.41	0.36	0.44	0.57	− 0.26	0.15	0.40	0.34	0.42	0.59
*p* value	< 0.001**	0.006**	0.002**	0.008**	0.028*	< 0.001**	0.001**	< 0.001**	< 0.001**	0.019*	< 0.001**	0.18	< 0.001**	0.001**	< 0.001**
Communication and physical heath	0.41	0.24	0.31	0.24	0.20	0.40	0.39	0.39	0.60	− 0.28	0.11	0.41	0.28	0.36	0.51
*p* value	< 0.001**	0.025*	0.003**	0.025*	0.062	< 0.001**	< 0.001**	< 0.001**	< 0.001**	0.01*	0.3	< 0.001**	0.01*	0.001**	< 0.001**
School wellbeing	0.27	0.11	0.27	0.14	0.15	0.35	0.38	0.28	0.55	− 0.004	− 0.02	0.31	0.23	0.30	0.33
*p* value	0.011*	0.3	0.01*	0.21	0.15	0.001**	< 0.001**	0.009**	< 0.001**	0.97	0.85	0.003**	0.031*	0.006**	0.002**
Social wellbeing	0.19	0.11	0.21	0.15	0.10	0.36	0.38	0.29	0.46	− 0.22	0.13	0.25	0.22	0.32	0.59
*p* value	0.07	0.33	0.048*	0.16	0.36	0.001**	< 0.001**	0.006**	< 0.001**	0.041*	0.22	0.018*	0.045*	0.003**	< 0.001**
Family health	0.41	0.39	0.35	0.26	0.34	0.32	0.34	0.41	0.41	− 0.41	0.15	0.55	0.59	0.57	0.40
*p* value	< 0.001**	< 0.001**	0.001**	0.016*	0.001**	0.002**	0.001**	< 0.001**	< 0.001**	< 0.001**	0.18	< 0.001**	< 0.001**	< 0.001**	< 0.001**
Feelings about functioning	0.36	0.50	0.30	0.35	0.21	0.22	0.27	0.30	0.22	− 0.28	0.21	0.38	0.38	0.44	0.30
*p* value	0.001**	< 0.001**	0.005**	0.001**	0.046*	0.04*	0.012*	0.005**	0.046*	0009**	0.047*	< 0.001**	< 0.001**	< 0.001**	0.004**
Access to service	0.26	0.19	0.21	0.21	0.16	0.22	0.22	0.06	0.44	− 0.16	0.12	0.06	0.048	0.2	0.39
*p* value	0.013*	0.072	0.054	0.048*	0.15	0.04*	0.045*	0.57	< 0.001**	0.14	0.28	0.56	0.66	0.06	< 0.001**

^*^ indicates *p* < 0.05; ** indicates *p* < 0.01

### Reliability—internal consistency, test–retest reliability, sensitivity, and concordance between self-report and proxy-report

For the participant self-report items, Cronbach’s *α* for the 5 subscales of CP-QoL-Teen (HK) ranged from 0.84 to 0.92 (Table 6). When reviewing the “Cronbach’s *α* if item is deleted,” all items were less than the overall Cronbach’s *α* suggesting good reliability. For the primary caregiver proxy-report items, Cronbach’s *α* for the 7 subscales ranged from 0.84 to 0.95 (Table 6). Nearly all items in the 7 subscales had the “Cronbach’s *α* if item is deleted” less than overall Cronbach’s *α* except several items demonstrated an improvement in overall reliability by 0.01 to 0.04 if specific item being deleted. For instance, the items of “the way they use their arms and hands” and “the way they use their legs” were 0.86 and 0.88 (overall Cronbach’s *α* = 0.85) in the subscale of “feeling about functioning”; the item of “how much pain does your teenager have” was 0.88 (overall Cronbach’s *α* = 0.84) in the subscale of “access to service”; the item of “how happy they are” was 0.89 (overall Cronbach’s *α* = 0.87) in the subscale of “social wellbeing”; and item of “ability to keep up physically with peers” was 0.93 (overall Cronbach’s *α* = 0.91) in the subscale of “school wellbeing.” The overall Cronbach’s *α* found in all subscales in self-report and proxy-report were > 0.8 suggesting excellent internal consistency. Test re-test reliability was assessed in twenty participants and their caregivers using convenience sampling. Moderate to excellent test–retest reliability were demonstrated in the 5 subscales with ICC coefficients ranging from 0.46 to 0.80 (self-report) and in the 7 subscales with ICC coefficients ranging from 0.40 to 0.72 (proxy-report) (Table 6). In terms of sensitivity, no floor effects were observed in the subscales of both self-report and proxy-report, whereas ceiling effects were observed in both proxy-report (ranged from 3.1 to 7.3%) and self-report (ranged from 0 to 12.5%). The finding suggested a moderate ceiling effect (12.5%) being observed in the subscale of self-reported feeling about functioning. Poor concordance between the subscale scores of self-report and proxy-report (ICC = 0.10 to 0.39) was identified with the lowest agreement observed in the subscale of “feelings about functioning” (Table 6).

**Table 6:** Internal consistency, sensitivity, and concordance between adolescents (self-report) and primary caregiver (proxy-report)

Subscale (no. of items)	Internal consistency		Sensitivity				Concordance between self-report and proxy-report (ICC)	Test–retest reliability	
	Self-report (*n* = 96)	Proxy-report (*n* = 91)	Self-report (*n* = 96)		Proxy-report (*n* = 91)			Self-report (*n* = 20)	Proxy-report (*n* = 20)
	Cronbach’s *α*	Cronbach’s *α*	Floor effect (*n*, %)	Ceiling effect (*n*, %)	Floor effect (*n*, %)	Ceiling effect (*n*, %)		ICC	ICC
General wellbeing and participation (21)	0.92	0.95	0 (0%)	1 (1%)	0 (0%)	5 (5.2%)	0.32**	0.80**	0.66**
Communication and physical health (16)	0.91	0.91	0 (0%)	0 (0%)	0 (0%)	5 (5.2%)	0.24*	0.77**	0.57**
School wellbeing (8)	0.87	0.91	0 (0%)	2 (2.1%)	0 (0%)	5 (5.2%)	0.30**	0.80**	0.56**
Social welling (7)	0.84	0.87	0 (0%)	3 (3.1%)	0 (0%)	5 (5.2%)	0.39**	0.46*	0.50**
Feelings about functioning (5)	0.87	0.85	0 (0%)	12 (12.5%)	0 (0%)	5 (5.2%)	0.10	0.65*	0.72**
Access to services (9)	N/A	0.84	N/A	N/A	0 (0%)	3 (3.1%)	N/A	N/A	0.40*
Family health (4)	N/A	0.88	N/A	N/A	0 (0%)	7 (7.3%)	N/A	N/A	0.61**

*ICC* intraclass correlation coefficient

^*^ indicates *p* < 0.05; ** indicates *p* < 0.01

## Discussion

The present study sets out to translate and evaluate the psychometric properties of the adolescent self-report and primary caregiver proxy-report versions of CP QoL-Teen (HK). In our translated version of CP QoL-Teen (HK), the Cronbach’s alpha varied from 0.84 to 0.92 in adolescents self-report in 5 subscales and ranged from 0.84 to 0.95 in primary caregiver proxy-report in 7 subscales. Despite some items of the proxy-report demonstrated “Cronbach’s *α* if item is deleted” would improve the reliability by 0.01 to 0.04, the differences were subtle and negligible. Hence, no item was considered as culturally irrelevant or redundancy for deletion. The overall Cronbach’s *α* in all subscales in self-report and proxy-report was > 0.8 suggesting an excellent internal consistency. The data was well matched with the original version (self-report: ranged from 0.78 to 0.96; proxy-report: 0.81 to 0.96) [7] and other translated versions [self-report: ranged from 0.75 to 0.92 [20, 21]; proxy-report: ranged from 0.79 to 0.94 [20, 21]] reported in different populations. These findings suggest an excellent internal consistency of the translated version of CP QoL-Teen (HK). Test–retest reliability of CP QoL-Teen (HK) (Self-report: ICC = 0.46 to 0.80; proxy-report: ICC = 0.40 to 0.72) fell within the range reported in original paper (self-report: ICC = 0.57 to 0.88; proxy-report: ICC = 0.29 to 0.83) [7] suggesting that CP QoL-Teen (HK), both self- and proxy- report versions, are reliable tool to assess the quality of life of adolescents.

Similar to the original English version [7], no floor effects were observed. However, CP QoL-Teen (HK) was found to have a higher ceiling effect in proxy-report (ranged from 3.1 to 7.3%) and self-report (ranged from 1 to 12.5%) than that reported original version [self-report: ranged from 1 to 4%; proxy-report: 4–7%] [7] and translated version [self-report: ranged from 0 to 1.3%; proxy-report: 0–4.7%] [20]. In particular, the moderate ceiling effect (12.5%) was found in the self-report subscale of “feelings about functioning,” which was in contrast with those reported by Power and co-workers, where the moderate ceiling effect of 13.6% was found in the same subscale but not in proxy-report subscale [20]. These findings suggest the good sensitivity of the CP QoL-Teen (HK).

In this study, the self-report and proxy-report version were distributed to the adolescents with CP and their primary caregivers. The proxy-report is particularly important when assessing the quality of life of children and adolescents who are too young or sick [9] despite inconsistent findings about concordance/disconcordance between self-report (children/adolescents) and proxy-report (parents) being reported [10].

Both original version of CP QoL-Teen (ICC = 0.4 to 0.61) [7] and translated version (ICC = 0.5 to 0.8) [20] demonstrated good concordance between the self-report and proxy-report by primary caregivers. However, our study reported poor agreement (ICC = 0.10 to 0.39) in all 5 subscales between self-report and proxy-report, particularly in the subscale of “feelings about functioning” (ICC = 0.10). Similar to other published studies in people with CP [22], we observed that the self-reported subscale scores of quality of life by adolescents were, in general, higher than that in the proxy-report (range from 2.4 to 9.3 unit, *p* < 0.05). In particular, the differences were in the subscales of “general wellbeing” (mean difference (MD) = 5.5 ± 16.2, *p* = 0.002) and “feelings of functioning” (MD = 9.3 ± 24.7, *p* < 0.001). Feeling, as non-observable behaviors, is usually one commonly reported discrepancy domain rather than those observable physical functioning [10]. Adolescents tended to perceive themselves to be more able to function than the perception of their parents’ [23]. Such discrepancies could be related to the increased age-associated independence [22], differences in reasoning, response styles, interpretations to the items being assessed [24], and difficulties to express feelings during adolescence [22]. The influence of gender, however, was not apparent in the result findings. We found no between-gender difference in all the subscale scores reported in both self- and proxy-format [10, 25]. In contrast, gender differences in quality of life scores were reported in the subscales of “general well-being” and “participation” of CP Q-L-Teen in India [26] and the subscale of “access to services” assessed in Bangladesh [20]. These suggest that the potential influence of socio-cultural context on gender equality could be an essential factor influencing the results. Further evaluation on how cultural, socio-economical, and gender equality may influence the quality of life of adolescents with CP and concordance between self- and primary caregiver (proxy-) report is needed. Such phenomena suggest that adolescents also play an essential role in disease management planning.

Subscales of CP QoL-Teen and subscales of CHQ, a generic HRQoL scale, and levels of GMFCS-E&R were compared to examine the concurrent validity. Both self-report and proxy-report of CP QoL-Teen (HK) were weakly to moderately correlated with some but not all of the subscales of CHQ-CF87 (*r*_s_: 0.21 to 0.61) and CHQ-PF50 (*r*_s_: − 0.41 to 0.60) indicating that both tools shared similar conceptual underpinning on general well-being. The disagreement on some subscales suggests the unique contribution of CP QoL-Teen (HK) in tapping on issues specifically addressing the conditions of cerebral palsy which would be unaddressed by generic health-related questionnaire, i.e., CHQ. Functionality and quality of life are distinct concepts [7, 27]. This also explains why associations were found between the subscale of “feelings about functioning,” but not in other subscales, and GMFCS-E&R in self-report (*r*_s_ = − 0.26, *p* = 0.011) and proxy-report (*r*_s_ = − 0.28, *p* = 0.008) suggesting the specificity of CP QoL-Teen (HK) subscale to detect the functional status of people with CP.

## Study limitation

The current study is limited by the small sample size despite extensive recruitment through normal schools and all special schools for children/adolescents with physical impairment. As the sample size was less than those reported sample size for factor analysis (*n* ≥ 200) [28, 29], confirmatory factor analysis and exploratory factor analysis were not conducted. The specific intelligence level of participants was not readily available for analysis in the current study. However, comparison between the data of participants taking normal curriculum versus adjusted curriculum may shed some light in this regard. In addition, our recruited participants were at GMFCS-E&R levels I to IV but not at level V. Hence, the lack of participants from level V may, potentially, affect its representativeness in this cohort of patients.

## Conclusion

The present study provided evidence that CP QoL-Teen (HK) has good psychometric properties. It is a reliable and valid tool in measuring the quality of life of adolescents with cerebral palsy in Hong Kong tailoring to the local cultural and social background.

## Data Availability

The datasets used during the current study are available from the corresponding author on reasonable request.

## Abbreviations

CP: Cerebral palsy
CP QoL-Teen: Cerebral Palsy Quality of Life Questionnaire-Teen
GMFCS-E&R: Expanded and revised version of Gross Motor Function Classification System
CHQ: Child Health Questionnaire
ICC: Intraclass correlation coefficient
HRQoL: Health-related quality of life
